# Phytoglobin: a novel nomenclature for plant globins accepted by the globin community at the 2014 XVIII conference on Oxygen-Binding and Sensing Proteins

**DOI:** 10.12688/f1000research.8133.1

**Published:** 2016-02-24

**Authors:** Robert Hill, Mark Hargrove, Raúl Arredondo-Peter

**Affiliations:** 1 Department of Plant Science, University of Manitoba, Winnipeg, Canada; 2 Molecular Biology Building, Deptartment of Biochemistry, Biophysics & Molecular Biology, Iowa State University, Ames, USA; 3 Laboratorio de Biofísica y Biología Molecular, Centro de Investigación en Dinámica Celular, Instituto de Investigación en Ciencias Básicas y Aplicadas, Universidad Autónoma del Estado de Morelos, Cuernavaca, Morelos, Mexico

**Keywords:** Algae, angiosperms, bryophytes, gymnosperms, legumes, nonsymbiotic, truncated

## Abstract

Hemoglobin (Hb) is a heme-containing protein found in the red blood cells of vertebrates. For many years, the only known Hb-like molecule in plants was leghemoglobin (Lb). The discovery that other Hb-like proteins existed in plants led to the term “nonsymbiotic Hbs (nsHbs)” to differentiate them from the Lbs. While this terminology was adequate in the early stages of research on the protein, the complexity of the research in this area necessitates a change in the definition of these proteins to delineate them from red blood cell Hb. At the 2014 XVIII Conference on Oxygen-Binding and Sensing Proteins, the group devoted to the study of heme-containing proteins, this issue was discussed and a consensus was reached on a proposed name change. We propose *Phytoglobin* (Phytogb) as a logical, descriptive name to describe a heme-containing (Hb-like) protein found in plants. It will be readily recognized by the research community without a prolonged explanation of the origin of the term. The classification system that has been established can essentially remain unchanged substituting Phytogb in place of nsHb. Here, we present a guide to the new nomenclature, with reference to the existing terminology and a phylogenetic scheme, placing the known Phytogbs in the new nomenclature.

Hemoglobin (Hb) is a heme-containing protein found in the red blood cells of vertebrates ^1^. Hemoglobin-like proteins are also found in other tissues of vertebrates where they are given tissue-specific names that help to identify their locations and distinguish them from red blood cell Hb ^2, 3^. For many years, the only known Hb-like molecule in plants was leghemoglobin (Lb), a protein induced as a result of the symbiotic relationship between legume plants and nitrogen-fixing bacteria ^4^. The discovery that other Hb-like proteins existed in plants not capable of symbiotic relationships led to the term “nonsymbiotic Hbs (nsHbs)” to differentiate them from the Lbs ^5^. While this terminology was adequate in the early stages of research on the protein, the complexity of the research in this area necessitates a change in the definition of these proteins to delineate them from red blood cell Hb, in keeping with the terminology for other Hb-like proteins, such as myoglobin in muscle, neuroglobin in neuron tissue and cytoglobin in vertebrate cell cytoplasm ^2, 3^. In 2001 Hunt *et al.* ^6^ classified plant Hbs as globin (GLB)0, GLB1, GLB2, GLb3 and GLBS corresponding to undetermined (mostly liverwort and moss) nsHbs, angiosperm nsHbs class/type 1 and nsHbs class/type 2, truncated Hbs and symbiotic Hbs (which included Lbs), respectively. However, an epithet for plant Hbs was absent in this nomenclature and distinctive characteristics for each category were not fully defined resulting in an incomplete classification system.

At the 2014 XVIII Conference on Oxygen-Binding and Sensing Proteins, the group devoted to the study of heme-containing proteins, the above issue was discussed and a consensus was reached on a proposed name change. *Phytoglobin* ( *phyto*, plant; *globin*, heme-containing protein folding structurally similar to the sperm whale myoglobin structure whose heme-Fe is invariably coordinated at the proximal site by His F8), abbreviated as Phytogb, was proposed as a logical, descriptive name to describe a heme-containing (Hb-like) protein found in plants. It will be readily recognized by the research community without a prolonged explanation of the origin of the term, as is the case for ‘nonsymbiotic hemoglobin’. The classification system that has been established can essentially remain unchanged substituting Phytogb in place of nsHb. A guide to the new nomenclature, with reference to the existing terminology, is given in Table 1. A more detailed phylogenetic scheme, placing the known Phytogbs in the new nomenclature, is shown in Figure 1. Also, we propose that acronym for the species-specific Phytogbs corresponds to the first three binomial (i.e. genus and species) letters followed by the Phytogb type and *phytogb* number of copy. For example, the acronym for rice ( *Oryza sativa*) Phytogb1.1 (see Table 1) corresponds to OrysatPhytogb1.1.

**Table 1.** System and characteristics of the accepted nomenclature for plant (algae + land plants) Phytoglobins (Phytogb).

Former plant globin name and abbreviation (in parenthesis)	New nomenclature ^a^	Plant origin	Distinctive characteristics ^b^
Nonsymbiotic hemoglobin (nsHb)	**Phytogb0**	Algae ^c^+bryophytes+ gymnosperms	Heme-Fe either penta- or hexacoordinate. Moderate to high affinity for O _2_. Localized in any plant organ.
Class/type 1 nonsymbiotic hemoglobin (nsHb-1)	**Phytogb1**	Angiosperms	Heme-Fe predominantly hexacoordinated by a distal amino acid. Extremely high affinity for O _2_ mostly due to a very low O _2_-dissociation rate constant ( *k*_off_). Localized in any plant organ.
Class/type 2 nonsymbiotic hemoglobin (nsHb-2)	**Phytogb2**	Angiosperms	Heme-Fe predominantly pentacoordinated. Moderate to high affinity for O _2_. Localized in any plant organ.
Symbiotic hemoglobin (symHb)	**SymPhytogb**	Non-legume N _2_-fixing plants ^d^	Heme-Fe predominantly pentacoordinated. Moderate to high affinity for O _2_. Specifically localized in N _2_-fixing nodules of actinorhizal plants or any other non-legume land plant
Leghemoglobin (Lb)	**Lb**	N _2_-fixing legumes ^d^	Heme-Fe predominantly pentacoordinate. Moderate to high affinity for O _2_. Specifically localized in legume N _2_-fixing nodules.
Class/type 3 nonsymbiotic hemoglobin/ Truncated hemoglobin (tHb)	**Phytogb3**	Algae ^c^+land plants	Globin-domain amino acid sequence and structure (i.e. folding into the 2/2-fold) similar to those of bacterial tHbs. Heme-Fe either penta- or hexacoordinate. Moderate to high affinity for O _2_. Localized in any plant organ.

^a^Numerical classification corresponds to that previously proposed by Hunt *et al.* ^6^. Proteins coded by multiple *phytogb* gene copy numbers within the same plant species should be indicated as the number of copy after the Phytogb numerical classification. For example, rice ( *Oryza sativa*) Phytogbs 1 and 2 (corresponding to the former nsHbs-1) should be indicated as rice Phytogb1.1 and Phytogb1.2, respectively (see text for a description on the species-specific Phytogbs acronym). ^b^Heme-Fe coordination and affinity for O _2_ correspond to those from moss Phytogb0 ^7– 9^, barley ^10^, rice ^11^ and Arabidopsis ^12^ Phytogb1, Arabidopsis Phytogb2 ^12^, *Casuarina* SymPhytogb ^13^, soybean Lb ^14, 15^ and Arabidopsis Phytogb3 ^16^ representative of Phytogb0, Phytogb1, Phytogb2, SymPhytogb, Lb and Phytogb3, respectively. ^c^Amino acid sequence of algal globins analyzed so far ^17– 19^ is similar to that of land plant Phytogb0 and Phytogb3, hence algal globins can be classified as Phytogb0 or Phytogb3, respectively. ^d^Some SymPhytogbs and Lbs (such as the *Parasponia* ^20^ and *Casuarina* ^21^ and *Chamaecrista* ^22^ globins, respectively) are intermediate between Phytogbs1 and Phytogbs2 and SymPhytogbs and Lbs ^22, 23^ because they exhibit amino acid sequence similarity to Phytogbs1 and Phytogbs2 ( Figure 1) and are localized in non-legume an legume nodules and apparently play a role in symbiotic N _2_-fixation.

**Figure 1.**
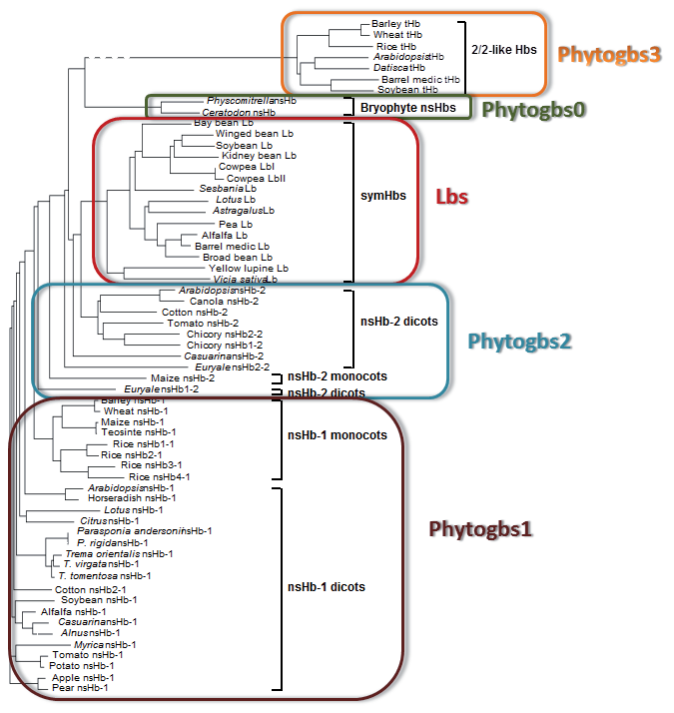
Phylogenetic representation of the novel nomenclature for land plant Phytogbs. Note that *Parasponia*, *Casuarina*, *Alnus* and *Myrica* SymPhytogbs are intermediate between SymPhytogbs and Phytogbs1 and Phytogbs2 (see Table 1 for explanation). Figure modified from Garrocho-Villegas *et al.* ^23^ (reprinted with permission).
