# Supplementary material for: Phytoglobin: a novel nomenclature for plant globins accepted by the globin community at the 2014 XVIII conference on Oxygen-Binding and Sensing Proteins
Source: F1000Res. 2016 Feb 24;5:212. [Version 1] doi: 10.12688/f1000research.8133.1 (PMC4792203; doi:10.12688/f1000research.8133.1)
Supplement: Peer review history

# Peer review: Referee response for version 1

**DOI:** 10.5256/f1000research.8749.r12637

**Published:** 2016-03-11

**Authors:** Kurt V Fagerstedt

**Affiliations:** 1 Department of Biosciences, University of Helsinki, Helsinki, Finland

The change in the hemoglobin/phytoglobin nomenclature presented in Hill *et al. *paper is timely and well supported by the hemoglobin and phytoglobin community. The change has been discussed in many conferences including the 2014 XVIII Conference on Oxygen-Binding and Sensing Proteins mentioned in the paper. The system presented in Table 1 is logical and supported by the protein structures and by the phylogenetic tree presented in Figure 2. The only and very slight problem is in the positioning of symbiotic hemoglobins present in non-legume nitrogen fixing plants, which have amino acid sequence similarities with both Phytogbs1 and 2. However, their positioning as a separate group in Table 1 is supported by the fact that they are only found in the nodules of actinorhizal plants.

Dr. Topunov has provided a historical perspective on the evolution of the name phytoglobin in his referee report, which clearly shows both the need for the term and no scientific discrepancy in its use. I agree wholeheartedly.

I have read this submission. I believe that I have an appropriate level of expertise to confirm that it is of an acceptable scientific standard.

# Peer review: Referee response for version 1

**DOI:** 10.5256/f1000research.8749.r12635

**Published:** 2016-03-08

**Authors:** Alexey F. Topunov

**Affiliations:** 1 Bach Institute of Biochemistry, Research Center of Biotechnology of the Russian Academy of Sciences, Moscow, Russian Federation

The reviewing article is dedicated to very interesting and actual issue. After proposition of the term “leghemoglobin” (Lb) by Virtanen and Laine (1946) ^1^ it was used only for hemoglobins (Hb) of the leguminous plants because it was the only one group of plants known to contain Hb. However after observing of Hbs in non-leguminous plants (Appleby *et al.* 1983 ^2^, Tjepkema 1983 ^3^) the ironic situation originated. “Non-leguminous” hemoglobins are evolutionary closer to leghemoglobins, but according to their denominations they looked to be similar to animal ones. Even hemoglobin of cyanobacteria received its own specific denomination “cyanoglobin” (Hill *et al* 1996) ^4^ although it was initially illogically named “Myogloblin in a cyanobacterium” (Potts *et al* 1992) ^5^, but plant Hbs were poor relatives in this family. The necessity of new terminology for plant hemoglobins appeared and such thoughts apparented and were discussed.  

The proposition of the new terminology “phytoglobin” (Phb) was already once stated. It was made in Bach Institute of Biochemistry (Moscow, Russia) in the book by Kretovich ^6^ and this term was even used in the published article (Topunov 1994) ^7^. This idea was not further developed at that time and in the next paper (Topunov 1995) ^8^ the old word “leghemoglobin” was employed again but using of the “phytoglobin” terminology in the article ^7^ was referred.

In conclusion:  it is the right desire to revive this terminology for plant hemoglobins and it has to be promoted and continued. There is the one difference between these two propositions of the “phytoglobin” term. In 1990s it was used as the general terminology for all plant hemoglobins (from both leguminous and non-leguminous plants) ^6^, ^7^ so “leghemoglobin” term could be excluded. Now it is proposed for plant hemoglobins aside from Lb, and it looks more pragmatic because scientists are accustomed to the “leghemoglobin” term and there is no need to exclude it from the scientific practice.

I have read this submission. I believe that I have an appropriate level of expertise to confirm that it is of an acceptable scientific standard.

## References

[1] VIRTANENAILAINET: Red, brown and green pigment in leguminous root nodules. *Nature*.1946;157:25 2101508710.1038/157025a0

[2] ApplebyCATjepkemaJDTrinickMJ: Hemoglobin in a nonleguminous plant, parasponia: possible genetic origin and function in nitrogen fixation. *Science*.1983;220(4600) : 10.1126/science.220.4600.951 951-3 10.1126/science.220.4600.951 17816020

[3] TjepkemaJD: Hemoglobin in the nitrogen fixing root nodules of actinorhizal plants. *Can J Bot*.1983;61(11) :2924-2929

[4] HillDRBelbinTJThorsteinssonMVBassamDBrassSErnstABögerPPaerlHMulliganMEPottsM: GlbN (cyanoglobin) is a peripheral membrane protein that is restricted to certain Nostoc spp. *J Bacteriol*.1996;178(22) :6587-98 893231610.1128/jb.178.22.6587-6598.1996PMC178546

[5] PottsMAngeloniSVEbelREBassamD: Myoglobin in a cyanobacterium. *Science*.1992;256(5064) :1690-1 160928110.1126/science.256.5064.1690

[6] KretovichWL: The Assimilation and Metabolism of Nitrogen in Plants [in Russian]. *Nauka, Moscow*.1987;

[7] TopunovAF: Function of phytoglobin in legume nodules and the regulation of oxygen conditions. *Appl Biochem Microbiol*.1994;30(1) :1-12

[8] TopunovAF: Leghemoglobin and its role in regulation of oxygen concentration in nitrogen-fixing legume nodules. *Biochemistry (Moscow)*.1995;60(1) :45-49

# Response

**Published:** 2016-03-08

**Authors:** Raul Arredondo-Peter

**Affiliations:** Universidad Autonoma del Estado de Morelos, Mexico

We thank Dr. Topunov for his evaluation and providing information that complements the contents of this article.
